# Sex Differences in the Incidence of Obesity-Related Gastrointestinal Cancer

**DOI:** 10.3390/ijms22031253

**Published:** 2021-01-27

**Authors:** Ji-Won Heo, Sung-Eun Kim, Mi-Kyung Sung

**Affiliations:** Department of Food and Nutrition, Sookmyung Women’s University, Seoul 04310, Korea; hjiwon1021@naver.com

**Keywords:** sex, obesity, estrogen, esophageal cancer, liver cancer, colorectal cancer

## Abstract

Cancer is the second leading cause of death worldwide, with 9.6 million people estimated to have died of cancer in 2018. Excess body fat deposition is a risk factor for many types of cancer. Men and women exhibit differences in body fat distribution and energy homeostasis regulation. This systematic review aimed to understand why sex disparities in obesity are associated with sex differences in the incidence of gastrointestinal cancers. Cancers of the esophagus, liver, and colon are representative gastrointestinal cancers, and obesity is a convincing risk factor for their development. Numerous epidemiological studies have found sex differences in the incidence of esophageal, liver, and colorectal cancers. We suggest that these sexual disparities are partly explained by the availability of estrogens and other genetic factors regulating inflammation, cell growth, and apoptosis. Sex differences in gut microbiota composition may contribute to differences in the incidence and phenotype of colorectal cancer. To establish successful practices in personalized nutrition and medicine, one should be aware of the sex differences in the pathophysiology and associated mechanisms of cancer development.

## 1. Introduction

Cancer is one of the leading causes of death worldwide, accounting for an estimated 9.6 million deaths in 2018 [1,2]. Lung, colorectal, prostate, stomach, and liver cancers are the most common cancer types in men, while breast, colorectal, lung, cervix uteri, and thyroid cancers are the most common cancer types in women. The World Health Organization (WHO) has reported that 30–50% of all cancer cases are preventable [1]. Obesity, tobacco use, exposure to viruses such as hepatitis B virus and human papilloma virus, UV radiation, and environmental pollutants have been the major targets of cancer prevention.

Evidence has shown that adiposity in adults is a convincing risk factor for many types of cancer, including cancers of the esophagus, pancreas, liver, colorectum, breast (postmenopausal), endometrium, and kidney [3]. Fat tissue, in association with immune cells, drives chronic and systemic inflammation, which causes genetic mutation that promotes abnormal cell proliferation, a key component of carcinogenesis [4]. Adipose tissue maintains a sufficient number of Treg cells to support anti-inflammatory activities in a healthy condition, whereas adipose tissue in the context of obesity exhibits an excess of proinflammatory Th1 cells and adipokines in association with excess proinflammatory cytokines. Obesity also leads to systemic metabolic dysregulation, which results in hyperinsulinemia, dyslipidemia, hyperglycemia, and constant oxidative stress, resulting in a cellular microenvironment favoring tumor growth [5].

The WHO has reported that the obesity rate has tripled since 1975 [6] and there are more than 1.9 billion overweight adults, among which 650 million adults have obesity. It is also important to note that the overall percentage of women with obesity (15%) is higher than that of men (11%), despite men and women showing similar overweight rates. The prevalence of severe obesity is also higher in women than in men [7]. This difference might be due to a rapid increase in the obesity rate in postmenopausal women and a longer life expectancy in women than in men. Interestingly, men and women exhibit distinctive body fat distributions, with such differences being mainly explained by the actions of gonadal hormones. However, with developments in genetic technology, it has been suggested that sex chromosomes and gene–gene interactions play important roles in energy metabolism [8]. The location of fat depots has received much attention in terms of explaining sex differences in the incidence of many diseases, including cancer. Thus, it is necessary to understand the actions and interactions of sex hormones and sex chromosomes in sex-specific body fat distribution, in association with obesity-related non-communicable diseases such as cardiovascular heart diseases, diabetes, and cancer. In this review, we summarize sexual disparities in fat deposition and distribution associated with sex differences in the incidence of gastrointestinal cancers, including esophageal cancer, liver cancer, and colorectal cancer.

## 2. Sex Differences in Adipose Tissue Distribution and Energy Metabolism

Obesity is defined as abnormal fat accumulation that creates a condition of systemic inflammation, which leads to a higher risk of non-communicable diseases. The fundamental cause of excessive fat accumulation is an imbalance between energy intake and energy expenditure. Macronutrients, including carbohydrates, proteins, and fat, supply energy and are required for basal metabolism and physical activity. Excessive calories supplied by macronutrients are converted to fatty acids and then stored as triacylglycerides (TG) in fat tissues distributed in different locations.

**Figure 1:**
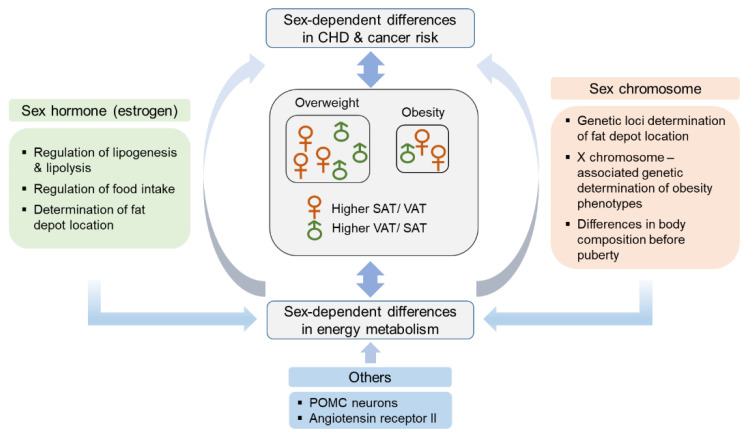
Factors contributing to sex differences in obesity. More women than men in the general population are obese, possibly because of a longer life expectancy in women and a rapid increase in obesity rate in postmenopausal women than in men. Sex differences in energy metabolism are contributed by not only sex hormone but also X chromosome gene expression, and expression of other genes, such as those for POMC and angiotensin receptor II. These factors regulate energy metabolism, leading to sex differences in the incidence of obesity and obesity-associated diseases. CHD, cardiovascular heart disease; POMC, pro-opiomelanocortin; SAT, subcutaneous adipose tissue; VAT, visceral adipose tissue.

Physiologically, women exhibit a higher tendency of deposition of fat in the form of subcutaneous adipose tissue (SAT), whereas in men, more fat tends to be deposited in the form of visceral adipose tissue (VAT), mostly distributed around abdominal organs [9]; VAT is known to be metabolically more active than SAT. Central and abdominal adipose tissue (subcutaneous upper body and visceral fat) are correlated with metabolic complications, while gluteal/femoral adipose tissue (lower body fat) has a lower metabolic risk [10]. VAT induces the elevation of circulating concentrations of insulin, free fatty acids (FFAs), and TG, while SAT shows lower lipolysis activity, thus posing a lower risk of metabolic complications. It has also been noted that VAT recruits proinflammatory substances that increase the risk of metabolic complications [11]. Adipose tissue is mostly composed of adipocytes, and adipose tissue mass is determined based on the number and size of adipocytes. Women tend to show an increase in fat mass based on an increase in the number and size of adipocytes, while men mostly show an increase in the size of adipocytes, predisposing them to systemic inflammation [12,13]. In addition, women have a higher amount of brown adipose tissue, which is involved in thermogenesis [14]. Sex differences in fat distribution and characteristics are determined by sex hormones, sex chromosomes, and other biological factors that have not yet been clearly identified (Figure 1).

### 2.1. Hormonal Factors

Androgens, estrogens, and progesterone are the major sex hormones produced in reproductive organs and various tissues. Estrogens play an important role in maintaining energy homeostasis. Rapid weight gain—especially an increase in abdominal fat mass—in postmenopausal women acts as evidence of the role of estrogens in energy homeostasis [15]. Estrogens, especially 17β-estradiol, regulate the expression of key genes involved in lipogenesis and lipolysis through sterol regulatory element binding protein-1c (SREBP-1c) and peroxisome proliferator-activated receptor (PPAR) γ [8].

Sex hormones participate in biological activities through nuclear receptors present in various tissues [16]. In the brain, estrogen receptor (ER)-α-bound 17β-estradiol controls food intake while maintaining energy homeostasis [17]. Studies have found that female animals show higher ER-α expression in many regions of the brain than their male counterparts [18,19] and that genetic deletion of ER-α affects female animals more than male animals [17]. Androgens have been shown to have a positive association with excess body weight in women [20], while an opposite effect was observed in men [21].

Estrogen also plays a pivotal role in sex-specific body fat distribution. Estrogen depletion leads to an increase in fat accumulation in the abdominal area, and estrogen repletion reverses this effect in female animals [22,23]. Estrogen-treated male animals have been reported to show decreased levels of visceral fat with an increase in subcutaneous fat levels compared to control animals [22]. It has been shown that SAT has higher levels of ERs and progesterone receptors than androgen receptors (AR) in female animals [24]. One multicenter clinical study showed that cross-sex hormonal therapy in trans women and trans men resulted in a more female-like body fat distribution in trans women and vice versa in trans men, emphasizing the role of hormones in body fat distribution [25]. Estrogen replacement therapy also decreases VAT mass in postmenopausal women [26].

Fat mass depends on the size of adipocytes, which is determined by TG accumulation through fatty acid metabolism; therefore, investigations on sex differences in fatty acid metabolism have been conducted. However, previous studies have shown that body adipose tissue lipolysis depends on the location of adipose tissue (upper body adipose tissue is more likely to show lipolysis than leg adipose tissue) and not on sex [27,28]. Insulin and exercise showed similar results in terms of leg adipose tissue lipolysis in both male and female individuals [29]. The storage pattern of dietary fatty acids was not different between men and women when they consumed an isocaloric diet; however, while consuming high-calorie and high-fat diets, women tended to store more fat in the form of lower body SAT than in the form of upper body SAT [30,31]. Few studies have been conducted to prove the effects of estrogens on fatty acid lipolysis or dietary fatty acid storage. However, one study found that postmenopausal women showed greater storage of dietary fatty acids than premenopausal women, although the mechanisms of action remained unclear [32].

### 2.2. Genetic Factors

The heritability of fat distribution measures has shown distinct differences between men and women [33]. Population studies on the heritability of anthropometric traits have indicated that the heritability of fat distribution measures is higher in women than in men [34]. Genome-wide association studies (GWAS) identified more than 100 loci associated with body fat distribution, represented by waist circumference (WC), hip circumference, and waist–hip ratio (WHR) [35,36,37]. Body mass index (BMI) was shown to be associated with 97 loci [36]. Among these loci, some showed sex differences, indicating that there are male- or female-specific biological pathways associated with obesity-related anthropometric measures. A meta-analysis of 32 GWAS found 14 loci significantly associated with WHR, and sex-specific analyses indicated that 14 loci were significant in women, while only three loci were significant in men. Another large-scale meta-analysis reported 49 loci associated with WHR [38], and 20 of the 49 loci revealed significant sex differences. In a rodent model, the analysis of adipose tissue gene expression in male and female animals fed with a high-fat diet revealed more than a few hundred differentially expressed genes [39,40].

All sex differences are primarily determined by sex chromosomes because sex chromosomes are the only chromosomes that differ between the male and female zygotes. The most important primary determinant of sex differences in the following downstream pathways is the *sry* gene, which causes the differentiation of testis in male zygotes, including the activation of genes that inhibit ovarian differentiation [41]. Therefore, *sry* is a key Y gene that determines testicular and ovarian development and regulates the secretion of testosterone in men and estrogen in women.

However, it has been noted that sex differences in body composition appear before gonadal hormone exposure. Male babies have a greater lean body mass and longer body length [42]. Lean body mass is greater in boys than in girls regardless of pubertal stage, and sexual dimorphism in fat patterning is apparent even in the pre-pubertal stage [43]. The possible functions of sex chromosomes, other than the functions related to the expression of gonadal hormones influencing fat deposition and distribution, had not been extensively investigated until the *sry* gene was used to generate a mouse model to test the pure effect of sex chromosomes [12]. In this model, the *sry* gene located in the Y chromosome was deleted in male mice to produce XY mice with ovaries. Additionally, the *sry* gene was translocated to a non-sex chromosome to generate XX mice with testis. These two models together with control XY mice with testis and XX mice with ovaries were used to differentiate the functions of sex hormone-producing gonads from the functions of sex chromosomes. In this initial experiment, the authors found that neuronal differences exist independent of gonadal hormones. In a later experiment using this four-core model system, mice were gonadectomized to remove the acute effects of gonadal hormones. The results indicated that obesity-associated phenotypes were positively associated with two X chromosome mice [44]. Thus, previous studies have aimed to identify the genes in the X chromosome that are responsible for body weight gain. The levels of X chromosome-associated genes, including *Eif2s3x*, *Kdm6a*, *Ddx3x*, *Kdm5c*, *Usp9x*, and *Uba1*, were higher in the fat tissue of XX mice than in the fat tissue of XY mice [44]. Other genes in the X chromosome shown to suppress food intake included the O-GlcNAc transferase (*Ogt*) gene [45] and the 5-HT2C receptor encoding *Ht2cr* gene [46].

Sex differences in energy homeostasis may be derived from factors other than sex chromosomes, and genes controlling food intake are representative examples. Pro-opiomelanocortin (POMC) is the pituitary precursor of many biologically active peptides, including melanocyte-stimulating hormone, corticotrophin (ACTH), and β-endorphin [47]. In the central nervous system, POMC-containing cell bodies often reside in the arcuate nucleus of the hypothalamus and the nucleus tractus solitarius of the brainstem, which regulate appetite and food intake [48]. Studies have found that POMC mRNA expression and neural activity are higher in female mice, thus leading to lower food intake in female mice [49]. TAp63, a transcription factor, and Sirt1 in POMC neurons have been suggested as key regulators of energy homeostasis [50,51]. Mice lacking angiotensin II receptors are more prone to diet-induced obesity; however, this effect is only seen in female mice [52]. Deletion of the gene expressing lecithin cholesteric acyl transferase protects female mice from diet-induced obesity [53].

## 3. Sex Differences in the Incidence of Major Gastrointestinal Cancers in Association with Obesity

### 3.1. Sex Differences in Esophageal Cancer Incidence

Esophageal cancer (EC) is the eighth most common cancer, and one of the major causes of cancer death worldwide [1,54]. It has two main subtypes depending on its histological characteristics: esophageal squamous cell carcinoma and esophageal adenocarcinoma. Esophageal squamous cell carcinoma accounts for more than 85% of all cases of EC and is related to tobacco use, alcohol and hot beverage consumption, and low intake of fruits and vegetables [1,3,55]. Although the incidence of esophageal squamous cell carcinoma has declined, the incidence of esophageal adenocarcinoma is increasing in Western countries, including the U.S.A. Overweight or obesity, gastroesophageal reflux disease, Barrett’s esophagus (BE), and tobacco use are risk factors for the development of esophageal adenocarcinoma [3,54]. Considering that obesity also increases the risk of gastroesophageal reflux disease and BE, the obesity epidemic is closely associated with the increased incidence of esophageal adenocarcinoma globally [2,56,57,58]. A meta-analysis from 25 epidemiological studies revealed that obesity increased the risk of EC, particularly esophageal adenocarcinoma [59]. There is a strong positive relationship between higher BMI and the risk of esophageal adenocarcinoma [60,61]. A pooled analysis reported a 2.4–4.8-fold increased risk of esophageal adenocarcinoma in individuals with a BMI ≥ 30 kg/m^2^, compared with that in individuals with a BMI < 25 kg/m^2^ [60].

Several studies have found that abdominal obesity, independent of BMI, is consistently associated with an increased risk of BE and esophageal adenocarcinoma [62,63]. Abdominal obesity is more common in men, which partly explains the higher incidence of esophageal adenocarcinoma in men than in women [1,9,64]. Abdominal obesity mainly involves the accumulation of VAT, which induces metabolic alterations, including alterations in the levels of insulin-like growth factor 1 (IGF-1) and adipokines such as leptin [10]. Human esophageal adenocarcinoma cell lines such as OE33, but not esophageal squamous cell carcinoma cell lines, showed increased proliferation in response to IGF-1 exposure [65]. In this study, the serum IGF-1 concentration was increased in patients with esophageal adenocarcinoma and EC patients with visceral obesity, suggesting that visceral and abdominal obesity might influence the progression of esophageal adenocarcinoma in association with IGF-1 levels [65]. In addition, diet-induced obesity was shown to increase the growth rate of esophageal tumors in OE33 tumor-bearing NOD-SCID mice, which was associated with increased levels of abdominal fat and serum leptin [66]. Leptin is involved in the regulation of food intake and energy homeostasis, and its level is elevated in the presence of obesity [67]. Leptin stimulates the growth of cancer cells, including esophageal adenocarcinoma cell lines, through the activation of janus kinase 2 (JAK2) and p38 mitogen-activated protein kinase (p38MAPK) pathways [68]. Interestingly, a case-control study reported that the serum leptin concentration is positively associated with the risk of BE in men but not in women [69]. Women show a higher ratio of subcutaneous leptin expression to visceral omental leptin expression than men, because leptin expression is predominant in SAT [70]. Given that there is an inverse relationship between serum leptin levels and BE incidence in women, despite higher levels of serum leptin than those in men, it is assumed that other factors, such as sex hormones, may contribute to sex differences in the incidence of esophageal diseases, including esophageal adenocarcinoma, which have a strong male predominance [1,64].

#### 3.1.1. Hormonal Factors

Accumulating evidence has shown an association between sex steroid hormones and esophageal adenocarcinoma. A nested case-control study revealed that high levels of serum estradiol, free estradiol, and dehydroepiandrosterone (DHEA) were correlated with a low risk of esophageal adenocarcinoma in men [71]. Similarly, a high ratio of androgens to estrogens was positively associated with esophageal adenocarcinoma risk in men [72]. In women, hormone replacement therapy reduced the risk of esophageal adenocarcinoma [73,74]. Thus, estrogen might play a role in the etiology of esophageal adenocarcinoma [75]. Estrogen increases not only leptin expression but also leptin sensitivity [23,76]. In addition, estrogen has anti-inflammatory functions, thus improving esophageal tissue damage, presumably by suppressing cytokine production via mast cell inactivation. [77,78]. A previous study showed that estrogen downregulates inflammation by inhibiting the expression of macrophage migration inhibitory factor (MIF), which is involved in innate and acquired immunity and cell growth [79,80]. Another animal study also found that estrogen treatment significantly decreased MIF expression in esophageal tissue [78]. In addition to its anti-inflammatory activity, estrogen ameliorates esophageal mucosal injury by improving esophageal barrier function through an increase in the expression of tight junction proteins [78,81]. Taken together, it is assumed that estrogen has protective effects against the incidence and progression of esophageal adenocarcinoma. Indeed, it has been noted that dietary intake of phytoestrogens, including lignans, quercetin, and resveratrol, is beneficial for the prevention of EC [82]. Epidemiological studies performed in women or in both sexes are limited by the low incidence of esophageal adenocarcinoma in women. Further studies are needed to investigate EC incidence by sex and the related underlying mechanisms.

#### 3.1.2. Genetic Factors

Several studies have reported sex-specific genetic associations for the development of esophageal adenocarcinoma. A meta-analysis of four GWAS showed that the genetic association between BMI and esophageal adenocarcinoma was significant in women. Moreover, the genetic correlation between WHR and esophageal adenocarcinoma was significant in men, suggesting that the accumulation of VAT, which is more abundant in men than in women, is positively associated with EC incidence [83]. In addition, another genome-wide meta-analysis identified two female-specific and three male-specific loci associated with the risk of BE and esophageal adenocarcinoma, which could contribute to sex disparities in EC susceptibility [84].

### 3.2. Sex Differences in Liver Cancer Incidence

Liver cancer (LC) is the sixth most common cancer and the fourth most common cause of cancer death worldwide [1]. Hepatocellular carcinoma (HCC) accounts for 90% of all LC cases and is prevalent in men, with a male-to-female incidence ratio of 1.3–5.5:1 [85]. Risk factors for HCC include hepatitis B or C viral infection, alcohol consumption, aflatoxin exposure, and obesity [1,3,86]. Non-alcoholic fatty liver disease (NAFLD) and non-alcoholic steatohepatitis (NASH), which are also associated with obesity and metabolic disorders, increase the risk of HCC [87,88]. Another study reported that lifestyle risk factors such as alcohol consumption and obesity were closely related to the incidence of HCC in North America and Europe [89]. A meta-analysis of 11 cohort studies reported that individuals with obesity showed associations with LC risk (relative risk (RR) = 1.89, 95% confidence interval (CI): 1.51–2.36), with a higher RR of LC in men with obesity (RR = 2.42, 95% CI: 1.83–3.20) than in women with obesity (RR = 1.67, 95% CI: 1.37–2.03); however, only three of these studies adjusted their analyses for alcohol consumption [90]. In a European cohort study, the strongest association was found between WHR and HCC risk [91]. Several studies have reported a positive association between higher BMI and HCC incidence. Men showed a stronger association between higher BMI and HCC risk than women [92,93]; in some studies, the association between higher BMI and HCC risk was only observed in men [94,95]. In addition, visceral fat accumulation was found to be an independent risk factor for HCC recurrence in HCC patients with suspected NASH [96]. Overall, the strong positive relationship between obesity and HCC incidence in men might be explained by the sex disparities in visceral fat deposition. VAT induces the production of not only proinflammatory adipokines, such as leptin, tumor necrosis factor α (TNF-α), interleukin (IL)-6, and hypoxia-inducible factor 1, but also immune cell infiltration [97]. VAT also induces a decrease in adiponectin levels and stimulates the release of FFA, which leads to the development of NAFLD and HCC [97].

In general, the incidence and prognosis of NAFLD, NASH, and HCC show different trends according to age and sex. Among younger patients, the prevalence of NAFLD and NASH is higher in men than in women. However, in women, the prevalence of NAFLD gradually increases with age, and the prevalence of NASH is higher in women aged >50–60 years than in men aged >50–60 years [98,99]. In a retrospective study of 1110 patients diagnosed with HCC between 2008 and 2017 (23.5% women), women showed a significantly better prognosis than men [100]. In particular, women aged <65 years showed longer overall survival (OS) than men aged <65 years (18.3 vs. 11.2 months), while there was no significant difference in OS between women aged ≥65 years and men aged ≥65 years (15.5 vs. 15.7 months) [100]. Similarly, another study also demonstrated sex differences in the OS of female HCC patients by age. In 34,674 HCC patients (24% women), sex was a protective factor for OS in patients aged 18–44 years, while no significant difference was observed in sex and OS among patients aged >65 years [101].

#### 3.2.1. Hormonal Factors

It appears that sex discrepancies in the incidence of HCC and OS of HCC patients are associated with exposure to sex hormones such as estrogen and androgen. An animal study using the *kras*^V12^ transgenic zebrafish model found that male fish showed faster HCC development with more severe and advanced features than female fish. In this study, estrogen treatment inhibited HCC progression in both sexes while androgen treatment enhanced it [102]. A growing number of studies have reported the protective roles of estrogen in the development and progression of HCC. In a mouse model lacking the ability to produce estrogen, 17β-estradiol treatment decreased hepatic steatosis and increased fatty acid β-oxidation, suggesting that estrogen is involved in the regulation of hepatic lipid homeostasis [103]. Estrogen treatment reduces lung metastasis in rats with HCC by inhibiting the expression of IL-6 and hepatocyte growth factor [104]. It has also been demonstrated that estrogen suppresses HCC progression through ER-α-induced inhibition of the nuclear factor kappa-light-chain-enhancer of activated B cells, which in turn decreases invasion and proliferation and increases apoptosis [105]. ER-α stimulates protein tyrosine phosphatase receptor type O, which is associated with the inactivation of signal transducers and activators of transcription (STAT) 3; this partly explains the sex disparity in HCC risk [106].

Conversely, a nested case-control study revealed that plasma testosterone levels were positively related to the risk of HCC among men [107]. In addition, androgens have been shown to induce the development and progression of HCC. Androgen acts mainly via the AR, which is upregulated in HCC tissue compared to that in normal liver tissue. Transgenic mice of both sexes lacking hepatic AR showed attenuation of HCC development, suggesting that AR expression increases HCC risk possibly by increasing oxidative stress and DNA damage and reducing p53-mediated DNA repair and apoptosis [108]. In another zebrafish model, liver-specific *ar* knockout also inhibited early HCC development, and androgen treatment stimulated HCC growth only in fish not lacking liver-specific *ar*, indicating that androgen-AR signaling plays a crucial role in HCC development [109].

#### 3.2.2. Genetic Factors

There has been growing evidence that genetic factors also contribute to sex differences in HCC susceptibility. Glycine N-methyltransferase (GNMT), which inhibits Wnt signaling, is known to be downregulated in HCC [110]. A previous animal study using *Gnmt^−^/^−^* mice demonstrated that female mice showed an increased risk of HCC compared with male mice. In addition, male and female mice showed distinct gene expression profiles for HCC tissues. Several genes involved in the MAPK pathway were upregulated only in female *Gnmt^−^/^−^* mice [111]. Furthermore, a sex-stratified analysis has revealed sex-specific etiologically relevant genes and biological pathways in HCC tumors [112]. PPAR pathway enrichment was observed in women, whereas other signaling pathways, including the PI3K/AKT, epidermal growth factor receptor (EGFR), and IL-2 pathways, were enriched in men. This study also found that 24.3% of discovered germline variants differentially modulated HCC gene expression in a sex-specific manner, indicating sex differences in the etiology of HCC [112].

### 3.3. Sex Differences in Colorectal Cancer Incidence

Globally, colorectal cancer (CRC) is the third most common cancer in men and the second most common cancer in women. CRC is the second leading cause of cancer mortality in both sexes [113]. It is known that the intake of processed/red meat, alcohol consumption, low intake of fruits and starchy vegetables, and smoking are risk factors for CRC [1,3]. Furthermore, there is a growing body of evidence showing that obesity increases the risk of CRC development and progression [114,115,116,117]. Epidemiological studies have demonstrated that the association between obesity and CRC incidence is stronger in men than in women [118,119]. A meta-analysis also reported that obesity at an age of <20 years was more likely to increase the risk of CRC in adulthood in men than in women [120]. Similarly, overweight men, both in childhood and early adulthood (17–26 years), showed a 2.7-fold greater risk of colon cancer than those with a consistently normal body weight [121]. These findings suggest the importance of the timing of onset and duration of overweight and obesity in terms of CRC risk.

In addition, a Mendelian randomization study revealed that higher BMI was associated with increased CRC risk only in men, whereas a higher WHR was more closely correlated with CRC risk in women than in men [122]. WC, not BMI, was also positively related to CRC risk in postmenopausal women, which might be associated with VAT accumulation [123]. Indeed, a cross-sectional study found that visceral fat mass determined by computed tomography was positively associated with CRC incidence in postmenopausal women [124]. Studies have found that a higher percentage of VAT was strongly related to CRC risk in both sexes [125] or only in men [126]. A transcriptomic analysis of human VAT and SAT demonstrated that genes associated with cytokines including IL-6, IL-8, and chemokine (C-C motif) ligand 2; cell adhesion; and metabolic homeostasis (gene for IGF-1) were upregulated in VAT compared to those in SAT, and a larger fat area was related to enhanced stimulation of inflammatory pathways [127]. An in vitro study using visceral adipocytes from individuals with obesity and CRC found that visceral adipocytes induce immune dysfunctions, including an increase in proinflammatory factor levels and immunosuppressive signals, leading to the development of CRC [128].

Sex differences also exist in CRC survival. A meta-analysis revealed that female CRC patients had better OS than male patients [129]; notably, female patients aged <45 years [130]. Consistent with this finding, young women (<50 years) showed better OS than young men, while older women (≥65 years) showed poorer OS than their male counterparts [131]. Therefore, based on the sex discrepancies in CRC prognosis observed before and after menopause, estrogen might have beneficial effects on CRC risk.

#### 3.3.1. Hormonal Factors

Estrogen functions through binding with ERs, such as ER-α and ER-β. ER-α is involved in the activation of STAT, PI3K, and MAPK signaling pathways, thus increasing CRC risk [132]. In contrast, ER-β activation reduces cell proliferation and induces apoptosis [133]. ER-α is highly expressed in CRC tissues, while ER-β is common in normal colon tissues [132,134]. It has been noted that estrogen affects the risk of CRC depending on the stage of CRC development; estrogen enhances ER-β expression to inhibit colon tumorigenesis in the early disease stages, whereas in the late disease stages, it stimulates ER-α expression, resulting in tumor progression [132]. Thus, it appears that obesity-induced elevation in estrogen levels might have a protective effect on CRC risk through the activation of ER-β [114,132]. In addition, the administration of exogenous estrogens (hormone replacement therapy) shows a protective effect against CRC [135]. A transcriptomic study has suggested that estrogen regulates the colon environment in a state of high-fat diet-induced obesity in a sex-specific manner [136]. A high-fat diet disrupts clock genes and increases macrophage infiltration in both male and female mice, while it also promotes epithelial cell proliferation in male mice. ER-β activation reverses these alterations, indicating that estrogen might play a protective role against the risk of obesity-associated CRC [136].

#### 3.3.2. Genetic Factors

Sex-specific biological disparities have been reported in the location and molecular features of CRC. Proximal (right-sided) colon cancer, which is more common among women, is characterized by microsatellite instability, CpG island methylator phenotype^+^, *BRAF* mutations, and hereditary non-polyposis colorectal cancer. Meanwhile, distal (left-sided) colon cancer, which is more prevalent in men, is linked to chromosomal instability, *p53* mutations, EGFR/Wnt signaling, and familial adenomatous polyposis [137,138]. In patients with stage III CRC, survival after adjuvant therapy with 5-fluorouracil and leucovorin was influenced by the TP53 genotype only in women [139]. Furthermore, the polymorphisms of vascular endothelial growth factor and lncRNA prostate cancer non-coding RNA were positively associated with CRC risk only in women [140,141].

It has been reported that there are sex-specific associations between the colonic expression of clock and clock control genes and survival of CRC patients [142]. Circadian disruption is known to promote tumor growth and decrease survival in cancer patients [143]. In women, low *cry2* expression and high ER-β expression were associated with better survival, while low *vegf-a* gene expression in tumors was associated with longer survival in men [142].

#### 3.3.3. Gut Microbiota

There is growing evidence that dysbiosis of the gut microbiota is linked to CRC risk [114,144,145]. One study reported similarities in gut microbiota composition between obesity and CRC patients. The proportions of *Hafnia alvei* (*Proteobacteria phylum*) and *Akkermansia muciniphila* (*Verrucomicrobia phylum*), known as mucin degraders, increased in both patient groups. This result indicates that the microbiome in a state of obesity might be associated with inflammation and cell damage induction, thus leading to an increased risk of CRC development [146]. Another animal study also found that sex-specific interactions between an obesogenic diet and the microbiota contributed to CRC development by reprograming of the intestinal epigenomes [147]. Furthermore, sex-specific differences in gut microbiota have been reported in an animal model of high-fat diet-induced obesity. Among old rats, the proportions of *Akkermansia muciniphila* and *Desulfovibrio spp.* increased in response to a high-fat diet only in female rats, suggesting that sex dimorphisms in the composition of the gut microbiota may be related to sex differences in inflammation and colon tumorigenesis [148].

## 4. Conclusions

**Figure 2:**
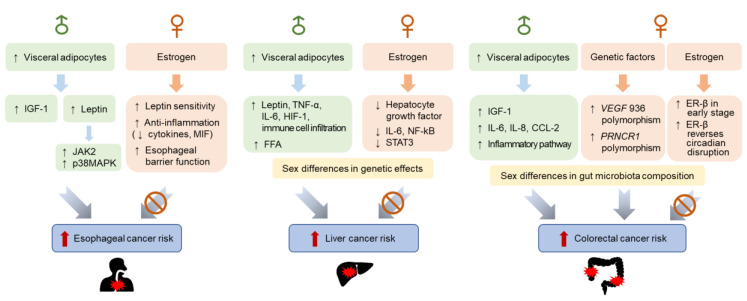
Sex differences in the incidence of major gastrointestinal cancers associated with obesity. Men and women exhibit differences in body fat distribution and energy homeostasis regulation. Overall, visceral adipose tissue, more abundant in men than in women, is positively associated with the incidence of esophageal, liver, and colorectal cancers. These sex disparities are partly explained by the availability of estrogens and other genetic factors regulating inflammation, cell growth, and apoptosis. Sex differences in gut microbiota composition may contribute to differences in the incidence and phenotype of colorectal cancer. CCL2, chemokine (C-C motif) ligand 2; ER-β, estrogen receptor β; FFA, free fatty acid; HIF-1, hypoxia-inducible factor 1; IGF-1, insulin-like growth factor 1; IL-6, interleukin 6; IL-8, interleukin 8; JAK2, janus kinase 2; MIF, macrophage migration inhibitory factor; NF-κB, nuclear factor kappa-light-chain-enhancer of activated B cells; p38MAPK, p38 mitogen-activated protein kinases; PRNCR1, lncRNA prostate cancer non-coding RNA; STAT3, signal transducers and activators of transcription 3; TNF-α, tumor necrosis factor α; VEGF, vascular endothelial growth factor.

Many epidemiological and experimental studies have indicated that there are sex differences in the incidence of cancer, including esophageal, liver, and colon cancers (Figure 2). Obesity is a common and highly convincing risk factor for many cancers, including major gastrointestinal cancers; therefore, sex disparities in body fat distribution and the regulatory mechanisms involved in energy homeostasis are suggested to contribute to sex differences in cancer incidence. The role of estrogen in body fat accumulation is the most well-understood mechanistic explanation for sex differences in obesity-associated cancers. Other genetic factors have been shown to contribute to sex differences in the incidence of major gastrointestinal cancer, although their association with obesity needs further investigation. The establishment of successful practices for personalized cancer prevention and treatment may require a clear understanding of sex-related biological differences in cancer risk.

## Abbreviations

AR	androgen receptor
BMI	body mass index
CI	confidence interval
CRC	colorectal cancer
EC	esophageal cancer
EGFR	epidermal growth factor receptor
ER	estrogen receptor
FFAs	free fatty acids
GWAS	genome-wide association studies
HCC	hepatocellular carcinoma
IGF-1	insulin-like growth factor 1
IL	interleukin
LC	liver cancer
MAPK	mitogen-activated protein kinase
MIF	migration inhibitory factor
NAFLD	non-alcoholic fatty liver disease
NASH	non-alcoholic steatohepatitis
OS	overall survival
MAPK	mitogen-activated protein kinases
POMC	pro-opiomelanocortin
PPAR	peroxisome proliferator-activated receptor
RR	relative risk
SAT	subcutaneous adipose tissue
STAT	signal transducers and activators of transcription
TG	triacylglyceride
VAT	visceral adipose tissue
WC	waist circumference
WHR	waist–hip ratio
